# Main protease of SARS-CoV-2 serves as a bifunctional molecule in restricting type I interferon antiviral signaling

**DOI:** 10.1038/s41392-020-00332-2

**Published:** 2020-10-06

**Authors:** Yaoxing Wu, Ling Ma, Zhen Zhuang, Sihui Cai, Zhiyao Zhao, Lingli Zhou, Jing Zhang, Pei-Hui Wang, Jincun Zhao, Jun Cui

**Affiliations:** 1 MOE Key Laboratory of Gene Function and Regulation, State Key Laboratory of Biocontrol, School of Life Sciences, Sun Yat-sen University, 510275 Guangzhou, Guangdong China; 2 State Key Laboratory of Respiratory Disease, Guangzhou Institute of Respiratory Disease, The First Affiliated Hospital of Guangzhou Medical University, 510182 Guangzhou, Guangdong China; 3 Advanced Medical Research Institute, Cheeloo College of Medicine, Shandong University, 250012 Jinan, Shandong China

**Keywords:** Innate immunity, Infectious diseases, Infection

**Dear Editor,**

At present, the world is suffering from an ongoing pandemic of 2019 novel coronavirus (COVID-19) which is caused by severe acute respiratory syndrome coronavirus 2 (SARS-CoV-2/2019-nCoV). To date, >20 million cases were confirmed with a death toll at >700,000. Although there are no clinically specific and effective antiviral treatments toward SARS-CoV-2 infection so far, the pathological study of SARS-CoV-2 infection and the development of SARS-CoV-2-specific vaccines are progressing rapidly within these several months.^1^ However, few reports mentioned the mechanism employed by SARS-CoV-2 for evading from surveillance of immune system.

During viral infection, type I interferon (IFN) responses serve as the first defensive line against invading viruses by inducing a group of antiviral interferon-stimulated genes (ISGs). Previous studies showed that early and proper type I IFN production could induce antiviral responses and potentiate the adaptive immune, thus effectively limiting coronavirus infection, including SARS-CoV-2.^1^ It has been reported that SARS-CoV-2 fails to induce robust IFN signaling,^2^ and impaired IFN responses was observed in patients with severe SARS-CoV-2 infection,^3^ suggesting that SARS-CoV-2 might develop multiple strategies to limit competent IFN production.

To investigate the influence of SARS-CoV-2 on type I IFN signaling during infection, we infected Huh7 cells and Calu3 cells with SARS-CoV-2 (Accession number: MT123290). SARS-CoV-2 infection induced type I IFN activation and enhanced the phosphorylation level of TANK-binding kinase 1 (TBK1) and IFN regulatory factor 3 (IRF3), leading to the induction of retinoic acid-inducible gene I (RIG-I), the major viral RNA sensor in the cytosol (Fig. 1a and Supplementary Fig. 1a–c). We next confirmed that RIG-I is required for *IFNB* induction by SARS-CoV-2 infection (Supplementary Fig. 1d). Although IFNβ pretreatment was shown to reduce the replication of SARS-CoV-2 effectively (Supplementary Fig. 1e) as previously reported,^2^ SARS-CoV-2-induced IFNβ signaling was relatively low (Supplementary Fig. 1f), suggesting that SARS-CoV-2 inhibited type I IFN production. We next ectopically expressed different SARS-CoV-2 proteins to study their roles in type I IFN signaling. Among them, SARS-CoV-2 main protease (M^pro^, also called 3CL^pro^ or nsp5) was proved to be a potent inhibitor of type I IFN signaling (Supplementary Fig. 1g). To confirm whether M^pro^ inhibits viral RNA-induced IFN signaling, we treated the M^pro^-transfected cells with intracellular poly(I:C) and found that M^pro^ reduced IFNβ signaling activation (Fig. 1b). As IFNβ induction requires coordination between IRF3- and nuclear factor (NF)-κB-mediated signaling pathways, we sought to determine the inhibition of M^pro^ in both IRF3-mediated and NF-κB pathway using IFN-stimulated response element (ISRE, which only needs IRF3 activation) and NF-κB luciferase reporters separately. M^pro^ was shown to inhibit both ISRE- and NF-κB-mediated signaling while the regulatory roles of M^pro^ in type I IFN pathway were relatively stronger (Supplementary Fig. 1h, i). Consistently, overexpression of M^pro^ could restrain the phosphorylation of TBK1 and IRF3 after Sendai virus infection (Supplementary Fig. 1j). We then found that M^pro^ decreased the IFNβ luciferase reporter activity induced by the active mutant of RIG-I [RIG-I (2CARD)] but not the downstream signaling proteins such as mitochondrial antiviral signaling protein, TBK1, or the active form of IRF3 [IRF3(5D)] (Fig. 1c). In addition, co-immunoprecipitation analysis showed that M^pro^ could interact with RIG-I but not the downstream signaling proteins (Fig. 1d), indicating that M^pro^ might target RIG-I. After recognizing viral RNAs, RIG-I undergoes K63-linked poly-ubiquitination mediated by TRIM25 to turn into its activated form by releasing its CARD domains. We next investigated whether M^pro^ affects the K63-linked ubiquitination of RIG-I as well as its association with its E3 ligase TRIM25.^4^ We found that overexpression of M^pro^ reduced the K63-linked ubiquitination of RIG-I as well as the interaction between RIG-I and TRIM25 after viral infection (Fig. 1e and Supplementary Fig. 1k, l). Taken together, these data revealed that SARS-CoV-2 M^pro^ might restrict IFN induction by reducing K63-linked ubiquitination on RIG-I. It remains elusive whether SARS-CoV-2 uses different strategies to suppress IFN signaling to improve its infectious ability, compared with SARS-CoV, another highly related pathogenic coronavirus. To investigate the differences between the M^pro^ of SARS-CoV-2 and SARS-CoV, we compared the function of M^pro^ from these two coronaviruses in regulating IFN signaling and found that M^pro^ of both coronaviruses could inhibit the IFN induction, while SARS-CoV-2 M^pro^ showed a relatively higher inhibitory activity than SARS-CoV M^pro^ (Fig. 1f). The stronger IFN antagonism of SARS-CoV-2 M^pro^ might further reduce antiviral responses in infected cells and thus enhancing SARS-CoV-2 incubation period and viral replication during infection.

**Fig. 1:**
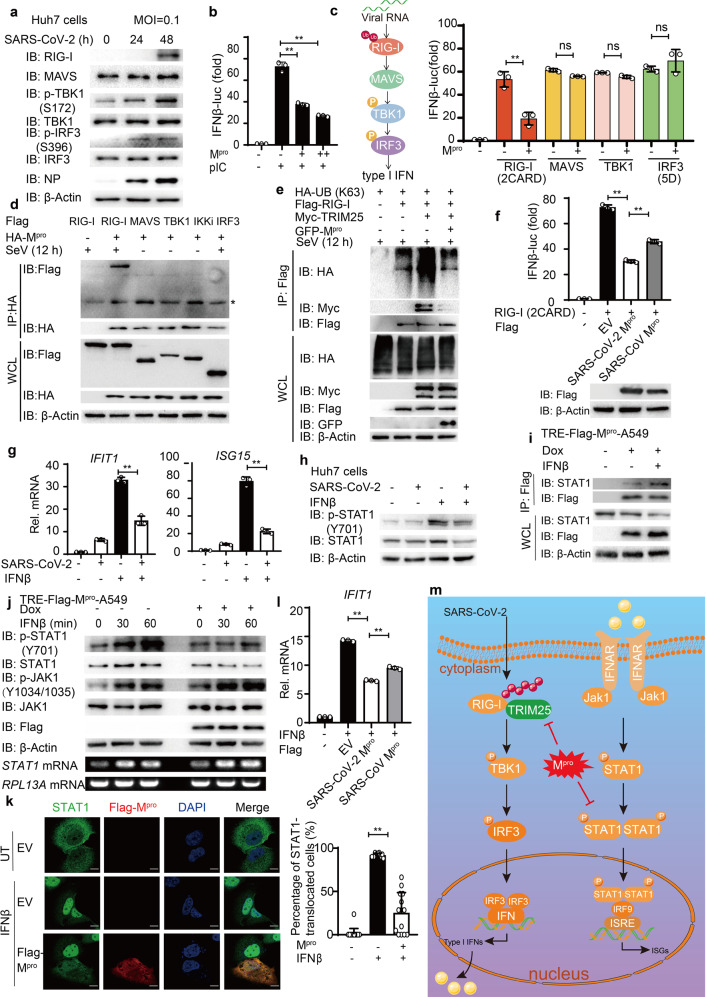
Diverse mechanisms utilized by SARS-CoV-2 M^pro^ in antagonizing type I IFN production and JAK-STAT signaling. **a** Immunoblot analysis of extracts of Huh7 cells infected with SARS-CoV-2 (MOI = 0.1) for the indicated time points. **b** Luciferase activity in 293T cells transfected with IFNβ luciferase reporter, together with empty vector (EV) or increasing amounts of M^pro^ of SARS-CoV-2. Then the cells were transfected with poly(I:C) for 12 h. **p < 0.01. **c** Luciferase activity in 293T cells transfected with IFNβ luciferase reporter and vectors for RIG-I (2CARD), MAVS, TBK1, and IRF3 (5D), along with empty vector or expression vectors for M^pro^. **d** 293T cells were transfected with plasmids encoding HA-M^pro^ and Flag-tagged key proteins in type I IFN signaling (Flag-RIG-I, Flag-MAVS, Flag-TBK1, Flag-IKKi, and Flag-IRF3) and treated with SeV (MOI = 0.1) for 12 h, followed by immunoprecipitation with anti-Flag beads and immunoblot analysis with anti-HA. Asterisk (*) indicates the nonspecific bands. **e** Lysates of 293T cells transfected with Flag-RIG-I, Myc-TRIM25, and HA-ubiquitin (K63 only) together with empty vector or expression vectors for M^pro^, followed with SeV infection (MOI = 0.1) for 12 h, were immunoprecipitated after SDS denaturation with anti-Flag and immunoblotted with the indicated antibodies. **f** Luciferase activity (above) in 293T cells transfected with IFNβ luciferase reporter and RIG-I (2CARD) along with empty vector or expression vectors for M^pro^ of SARS-CoV-2 and SARS-CoV. **p < 0.01. Immunoblot analysis (below) of extracts of 293T cells transfected with expression vectors for M^pro^ of SARS-CoV-2 and SARS-CoV. **g** Quantitative PCR with reverse transcription analysis of *IFIT1* and *ISG15* mRNA in Huh7 cells infected with SARS-CoV-2 (MOI = 0.1) for 24 h, followed by IFNβ treatment (1000 U ml^−1^) for 3 h. **p < 0.01. **h** Immunoblot analysis of extracts of Huh7 cells infected with SARS-CoV-2 (MOI = 0.1) for 48 h, followed with IFN treatment for 30 min. **i** Co-immunoprecipitation and immunoassay of extracts of M^pro^-inducible A549 cells were treated with doxycycline (Dox; 200 ng ml^−1^) for 24 h, followed by IFNβ treatment (1000 U ml^−1^) for 3 h. **j** Immunoassay of extracts of M^pro^-inducible A549 cells were treated with doxycycline (Dox; 200 ng ml^−1^) for 24 h, followed by IFNβ treatment (1000 U ml^−1^) for the indicated time points. Below, RT-PCR analysis of *STAT1* mRNA; *RPL13A* mRNA serves as a loading control. **k** Confocal microscopic analysis (left) of STAT1 localization in Huh7 cells transfected with empty vector or Flag-M^pro^ for 24 h, followed by IFNβ treatment (1000 U ml^−1^) for 30 min or left untreated (UT). Scale bars, 10 μm. Quantitative analysis (right) of the colocalization (30 cells per sample). **l** Quantitative PCR with reverse transcription analysis of *IFIT1* mRNA in Huh7 cells transfected with empty vector or expression vectors for M^pro^ of SARS-CoV-2 and SARS-CoV for 36 h, followed by IFNβ treatment (1000 U ml^−1^) for 3 h. **p < 0.01. **m** Schematic representation of SARS-CoV-2 M^pro^ antagonizing antiviral immunity. All the experiments are representatives of three independent biological experiments with similar results

Once secreted, type I IFN could bind to IFN receptor to induce the transcription of hundreds of ISGs through signal transducer and activator of transcription factor (STAT) proteins, to establish the antiviral state of the cells.^4^ We found that SARS-CoV-2 infection reduced the mRNA abundance of IFN-stimulated downstream cytokines (Fig. 1g) as well as IFN-triggered phosphorylation level of STAT1 (Fig. 1h), which suggested that SARS-CoV-2 could employ additional mechanisms to counter Janus-activated kinase (JAK)-STAT1 signaling. We next investigated whether SARS-CoV-2 M^pro^ could affect the induction of antiviral ISGs through JAK-STAT signaling. Co-immunoprecipitation analysis showed that SARS-CoV-2 M^pro^ could interact with STAT1 (Fig. 1i and Supplementary Fig. 2a). Ectopic expression of SARS-CoV-2 decreased the protein levels of STAT1 and impaired the IFN-induced phosphorylation of STAT1 and the nuclear translocation of STAT1 (Fig. 1j, k). Pharmacologic approaches showed that M^pro^-mediated degradation of STAT1 could be rescued by autophagy/autolysosome inhibitor 3-methyladenine and bafilomycin A1 (Supplementary Fig. 2b). Moreover, M^pro^ could promote the colocalization between STAT1 and microtubule associated protein 1 light chain 3 beta (LC3B) (Supplementary Fig. 2c), suggesting that M^pro^ might prompt the autophagic degradation of STAT1. IFN-triggered inductions of downstream ISGs were decreased in SARS-CoV-2 M^pro^-overexpressing cells (Supplementary Fig. 2d). We also compared the function between M^pro^ of SARS-CoV-2 and SARS-CoV on JAK-STAT signaling. M^pro^ from both coronaviruses could inhibit IFN-triggered inductions of *IFIT1* and promote the degradation of STAT1 (Fig. 1l and Supplementary Fig. 2e). Since M^pro^ functions as a protease, we also investigated whether M^pro^ restricted JAK-STAT signaling through its enzymatic activity by generating M^pro^ enzymatic inactive mutant C145S (CS). Our results revealed that M^pro^ CS mutant failed to reduce IFN-triggered ISG induction or enhance the association between STAT1 and autophagic receptor p62 (Supplementary Fig. 2f–g), suggesting that the enzymatic activity might be necessary for M^pro^ to inhibit JAK-STAT signaling. We next found that overexpression of SARS-CoV-2 M^pro^ reduced virus-triggered IFN production as well as downstream ISGs and enhanced viral replication during SARS-CoV-2 infection (Supplementary Fig. 2h, i). Taken together, our results showed that SARS-CoV-2 M^pro^ could inhibit both IFN production and JAK-STAT signaling to antagonize innate antiviral immunity, thus enhancing the viral replication and latency.

To date, the mechanisms used by SARS-CoV-2 to evade intracellular innate immune surveillance remains largely unknown. Our finding revealed the dual function of M^pro^, the main protease of SARS-CoV-2, in impairing both virus-triggered type I IFN production and the downstream ISG induction (Fig. 1m). M^pro^ is the key enzyme of coronaviruses that exhibits similar proteasome functionality by digesting at least 11 conserved sites of the viral polyproteins encoding the first open reading frame (ORF; ORF 1a/b) to process the polyproteins into multiple functional non-structural proteins during infection. As M^pro^ plays pivotal roles in mediating viral replication and transcription of SARS-CoV-2, several groups have designed drugs that target SARS-CoV-2 M^pro^ to cure COVID-19.^5^ By revealing the additional functions of SARS-CoV-2 M^pro^, our finding may shed new light on understanding the immune-evading mechanisms of SARS-CoV-2, which could provide novel targets for potential therapeutic intervention on SARS-CoV-2 infection.

## Supplementary information

Supplementary information
