# Supplementary material for: Main protease of SARS-CoV-2 serves as a bifunctional molecule in restricting type I interferon antiviral signaling
Source: Signal Transduct Target Ther. 2020 Oct 6;5:221. doi: 10.1038/s41392-020-00332-2 (PMC7537955; doi:10.1038/s41392-020-00332-2)
Supplement: Supplementary file 1 — Supplementary information [file 41392_2020_332_MOESM1_ESM.docx]

Supplementary Materials for

Main protease of SARS-CoV-2 serves as a bifunctional molecule in restricting type I interferon antiviral signaling

Yaoxing Wu^1#^, Ling Ma^1#^, Zhen Zhuang^2#^, Sihui Cai^1#^, Zhiyao Zhao^1^, Lingli Zhou^1^, Jing Zhang^3^, Pei-Hui Wang^3^, Jincun Zhao^2*^ and Jun Cui^1*^

^1^ MOE Key Laboratory of Gene Function and Regulation, State Key Laboratory of Biocontrol, School of Life Sciences, Sun Yat-sen University, Guangzhou, Guangdong 510275, China

^2^ State Key Laboratory of Respiratory Disease, Guangzhou Institute of Respiratory Disease, The First Affiliated Hospital of Guangzhou Medical University, Guangzhou, Guangdong 510182, China

^3^ Advanced Medical Research Institute, Cheeloo College of Medicine, Shandong University, Jinan, Shandong 250012, China

^#^These authors contributed equally to this work.

^*^Correspondence to: [cuij5@mail.sysu.edu.cn](mailto:cuij5@mail.sysu.edu.cn) or [zhaojincun@gird.cn](mailto:zhaojincun@gird.cn)

**This PDF file includes:**

Materials and Methods

Supplementary Fig. S1 to S2

**Materials and Methods**

**Cell culture and reagents**

HEK293T (human embryonic kidney 293T) cells, A549 cells and Huh7 cells were cultured in DMEM medium (Gibco) with 10% (vol/vol) fetal bovine serum (Gibco) in a 5% CO_2_ incubator at 37 °C. Calu3 cells were cultured in MEM (Gibco) with 1% NEAA (Gibco), 1% SODIUM PYRUVATE (Gibco) and 10% (vol/vol) fetal bovine serum (Gibco) in a 5% CO_2_ incubator at 37 °C. Recombinant human IFNβ was purchased from Peprotech. Poly (I:C) (LMW) was purchased from Invivogen. Doxycycline (D9891), MG132 (C-2211-5MG), Bafilomycin A1 (H2714) and 3-methyladenine (M9281-100MG) were purchased from Sigma.

**Plasmids and antibodies**

Plasmids encoding SARS-CoV-2 proteins were chemically synthesized based on severe acute respiratory syndrome coronavirus 2 (Accession number: MT123290).^1^ We sub-cloned these viral proteins into pcDNA3.1 expression vector using standard molecular biology techniques. RIG-I (2CARD), MAVS, TBK1, IRF3 (5D) and IFNβ, ISRE, NF-κB luciferase reporter plasmids have been previously described.^2^ Other plasmids mentioned were acquired by the means of standard PCR techniques. Horseradish peroxidase anti-Flag (M2; A8592) and anti-β-actin (A1978) were purchased from Sigma. Horseradish peroxidase anti-hemagglutinin (HA; 12013819001) and anti-c-myc (MYC; 11814150001) were purchased from Roche Applied Science. Anti-RIG-I (3743), Anti-pTBK1 (5483), anti-TBK1 (3013), anti-pIRF3 (4947s), anti-Jak1 (3344), anti-pJak1 (3331), anti-pSTAT1(9167), were acquired from Cell Signaling Technology. Anti-IRF3 (sc-9082) and anti-STAT1 (sc-346) were from Santa Cruz Biotechnology. Anti-N (40588-T62) were from Sino Biological.

**siRNA Transfection**

Chemically synthesized 21-nucleotide siRNA duplexes were obtained from TranSheepBio and transfected using Lipofectamine RNAiMAX (Sigma) according to the manufacturer’s instructions. siRNA sequences were:

scramble (SCR) siRNA: 5'-UUCUCCGAACGUGUCACGUTT-3'

*DDX58 (RIG-I)* siRNA: 5'-GAAUUAUCCCAACCGAUAUTT-3'

*IFIH1 (MDA5)* siRNA: 5'-GUAACAUUGUUAUCCGUUATT-3'

**Virus infection**

Cells were either mock-infected or infected with SARS-CoV-2 (Accession number: MT123290), as previously described.^1^ Virus was allowed to adsorb at 37 ^o^C for 1 hour with gentle rocking every 15 min before incubation at 37 °C for indicated time.^3^ SeV was kindly provided by Dr. F-Xiao-Feng Qin (Suzhou Institute of Systems Medicine). Cells were infected at different MOI and time points as indicated.

**Focus forming assay (FFA)**

Vero E6 cells were seeded in 96-well plates one day before infection. Virus was serially diluted and used to inoculate Vero E6 cells at 37°C for 1 hour. Inocula were then removed before adding 125 μl 1.6% carboxymethylcellulose warmed to 37°C per well. After 24 hours, cells were fixed with 4% paraformaldehyde and permeabilized with 0.2% Triton X-100. Cells were then incubated with a rabbit anti-SARS-CoV-2 nucleocapsid protein polyclonal antibody (40143-T62, Sino Biological), followed by an HRP-labelled goat anti-rabbit secondary antibody (109-035-088, Jackson ImmunoResearch Laboratories). The foci were visualized by TrueBlue Peroxidase Substrate (KPL, Gaithersburg, MD), and counted with an ELISPOT reader (Cellular Technology Ltd. Cleveland, OH). Viral titers were calculated as FFU per ml.

**Luciferase and reporter assays**

293T (2×10^5^) cells were plated in 24-well plates and transfected using Lipofectamine 2000, with plasmids encoding an IFNβ luciferase reporter (firefly luciferase; 100 ng) and pRL-TK (renilla luciferase plasmid; 10 ng) together with 100 ng plasmid encoding Flag-RIG-I (2CARD), Flag-MAVS, Flag-TBK1, or Flag-IRF3 (5D) and indicated variety expression plasmid of viral proteins or empty vector (pcDNA3.1) plasmid. Empty pcDNA3.1 vector was used to maintain equal amounts of DNA among wells. Then, the cells were infected with SeV or transfected with poly (I:C) for indicated time points. Cells were collected and luciferase activity was measured with the Dual-Luciferase Assay (Promega) with a Luminoskan Ascent luminometer (Thermo Scientific), according to the manufacturer’s protocol. Data represent relative firefly luciferase activity normalized to renilla luciferase activity.

**Immunoprecipitation and immunoblot analysis**

For immunoprecipitation, whole-cell extracts were prepared after transfection or stimulation with appropriate ligands, followed by incubation overnight with anti-Flag or anti-hemagglutinin agarose gels (Sigma). Beads were then washed five times with low-salt lysis buffer (50 mM HEPES, 150 mM NaCl, 1mM EDTA, 10% glycerol, 1.5 mM MgCl_2_, and 1% Triton X-100), and immunoprecipitates were eluted with 2 × SDS Loading Buffer and resolved by SDS-PAGE. Proteins were transferred to PVDF membranes (Bio-Rad) and further incubated with the appropriate antibodies. Immobilon Western Chemiluminescent HRP Substrate (Millipore) was used for protein detection.

**RNA extraction and quantitative RT-PCR**

Total RNA was extracted from cells using the Trizol reagent (Invitrogen) according to the manufacturer’s instructions. For RT-PCR analysis, cDNA was generated with HiScript® II Q RT SuperMix for qPCR (+gDNA wiper) (Vazyme, R223-01) and was analyzed by quantitative real-time PCR using the 2×RealStar Green Power Mixture (GenStar). All data were normalized to *RPL13A* expression. Primer sequences were:

*IFNβ:* Forward: 5′-CCTACAAAGAAGCAGCAA-3′

Reverse: 5′-TCCTCAGGGATGTCAAAG-3′

*ISG15*: Forward: 5′-TCCTGGTGAGGAATAACAAGGG-3′

Reverse: 5′-GTCAGCCAGAACAGGTCGTC-3′

*IFIT2*: Forward: 5′-GGAGGGAGAAAACTCCTTGGA-3′

Reverse: 5′-GGCCAGTAGGTTGCACATTGT-3′

*IFIT1:* Forward: 5′-TCAGGTCAAGGATAGTCTGGAG-3′

Reverse: 5′-AGGTTGTGTATTCCCACACTGTA-3′

SeV *pRNA*: Forward: 5′-GACGCGAGTTATGTGTTTGC-3′

Reverse: 5′-TTCCACGCTCTCTTGGATCT-3′

SARS-CoV-2 *N protein* RNA:

Forward: 5′-GGGGAACTTCTCCTGCTAGAAT-3′

Reverse: 5′-CAGACATTTTGCTCTCAAGCTG-3′

*RPL13A*: Forward: 5′-GCCATCGTGGCTAAACAGGTA-3′

Reverse: 5′-GTTGGTGTTCATCCGCTTGC-3′.

**Generation of M^pro^-Inducible Cell Lines**

For M^pro^-inducible expression, lentiviral particles were produced by transfecting HEK293T cells with FG-EH-DEST-M^pro^-Puro, VSGV and D8.9. A549 cells expressing Teton-3G were infected by incubation with lentivirus-containing supernatant for 48 hours. Transduced cells were purified by puromycin selection as previously described.^4^

**Fluorescence microscopy**

Cells were cultured on Glass Bottom culture dishes (Nest Scientific) and directly observed as precious described.^4^ For examination by immunofluorescence microscopy, cells were fixed with 4% paraformaldehyde for 30 min, and then permeabilized in methyl alcohol for 10 min at −20°C. After washing with PBS for 3 times, cells were blocked in 5% goat serum for 1 hour, and then incubated with primary antibodies diluted in 5% goat serum overnight. The cells were washed and followed by a fluorescently labeled secondary antibody (CF568 Goat anti-Mouse IgG (H+L), Biotium; CF568 Goat anti-Rat IgG (H+L), highly cross-adsorbed, Biotium; Goat anti-Rabbit IgG (H+L) Highly Cross-Adsorbed Secondary Antibody, Alexa Fluor 488, Invitrogen). Confocal images were examined using a microscope (TCS SP8 STED 3X; Leica) equipped with 100×1.40 NA oil objectives. The images were processed for gamma adjustments using Leica AS Lite or ImageJ software (National Institutes of Health).

**Statistical analyses**

Data are represented as mean ± SEM unless otherwise indicated, and Student’s *t*-test was used for all statistical analyses with the GraphPad Prism 5 software. Differences between two groups were considered significant when *P* value was less than 0.05.

**References**

1 Sun, J. *et al.* Isolation of infectious SARS-CoV-2 from urine of a COVID-19 patient. *Emerg Microbes Infect* **9**, 991-993, doi:10.1080/22221751.2020.1760144 (2020).

2 Cui, J. *et al.* USP3 inhibits type I interferon signaling by deubiquitinating RIG-I-like receptors. *Cell Res* **24**, 400-416, doi:10.1038/cr.2013.170 (2014).

3 Wu, Y. *et al.* Zika virus evades interferon-mediated antiviral response through the co-operation of multiple nonstructural proteins in vitro. *Cell Discov* **3**, 17006, doi:10.1038/celldisc.2017.6 (2017).

4 Jin, S. *et al.* Tetherin Suppresses Type I Interferon Signaling by Targeting MAVS for NDP52-Mediated Selective Autophagic Degradation in Human Cells. *Mol Cell* **68**, 308-322 e304, doi:10.1016/j.molcel.2017.09.005 (2017).

**Supplementary Fig. S1**

**
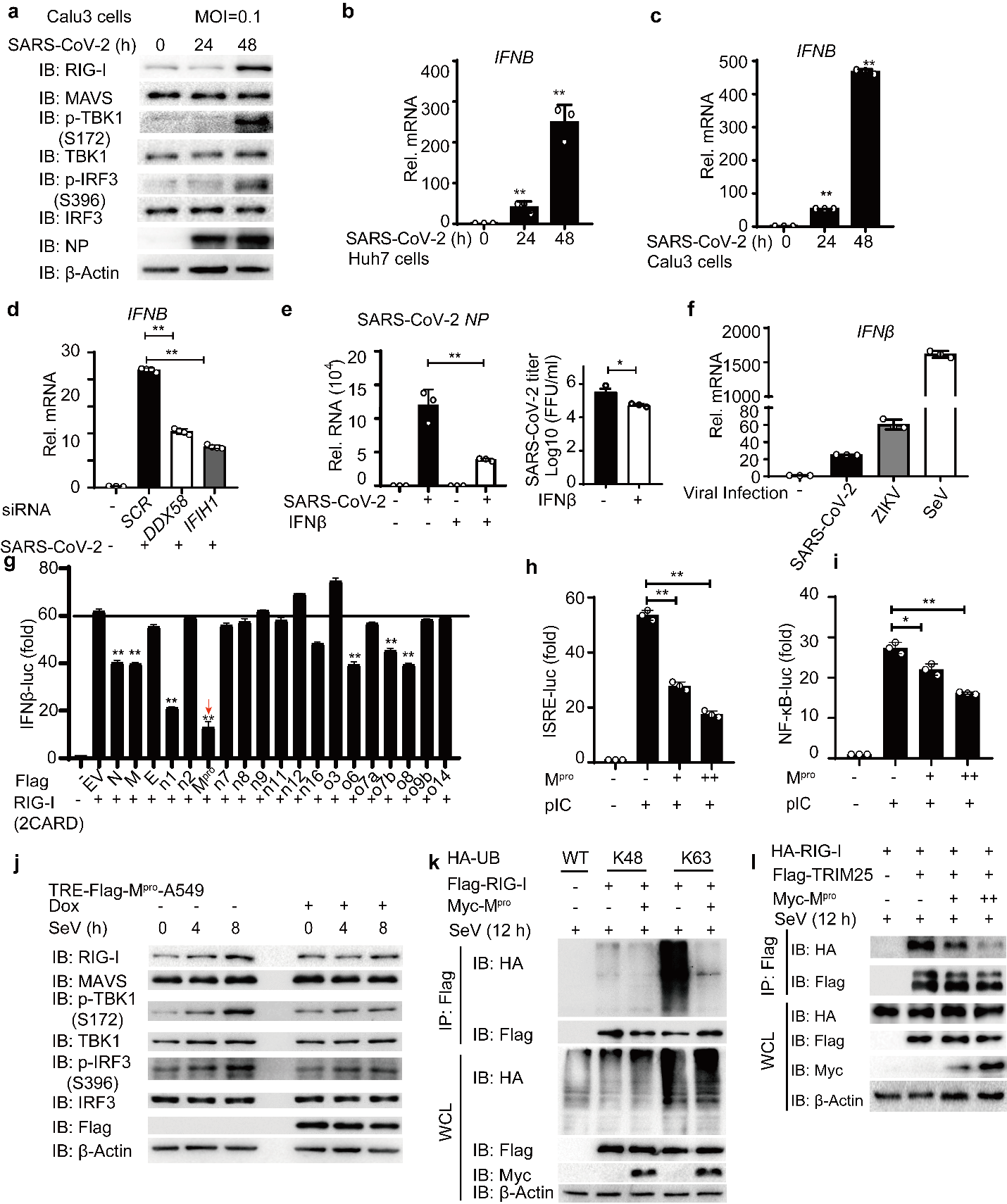
**

**Supplementary Fig. S1 SARS-CoV-2 M^pro^ antagonizes type I IFN induction by impairing K63-linked ubiquitination of RIG-I. a** Immunoblot analysis of extracts of Calu3 cells infected with SARS-CoV-2 (MOI=0.1) for indicated time points. **b-c** Quantitative PCR with reverse transcription analysis of *IFNB* mRNA in Huh7 cells (**b**) or Calu3 cells (**c**) infected with SARS-CoV-2 (MOI=0.1) for indicated time points. **p < 0.01. **d** Quantitative PCR with reverse transcription analysis of *IFNB* RNA in Huh7 cells transfected with scramble (SCR) siRNA, RIG-I-specific (*DDX58*) siRNA or MDA5-specific (*IFIH1*) siRNA followed with SARS-CoV-2 infection (MOI=0.1) for 24 hours. **p < 0.01. **e** SARS-CoV-2 replication determined by quantitative PCR (left) and Viral titration (right). (left) Quantitative PCR with reverse transcription analysis of SARS-CoV-2 *N protein* RNA in Huh7 cells with IFN pre-treatment (1000 U·ml^−1^) for 3 hours followed with SARS-CoV-2 infection (MOI=0.1) for 48 hours. **p < 0.01. (right) Viral titration of SARS-CoV-2 in the supernatant of Huh7 cells with IFN pre-treatment (1000 U·ml^−1^) for 3 hours followed with SARS-CoV-2 infection (MOI=0.1) for 48 hours using FFA. *p < 0.05. **f** Quantitative PCR with reverse transcription analysis of *IFNB* mRNA in Huh7 cells infected with SARS-CoV-2 (MOI=0.1), ZIKV (MOI=1) or SeV (MOI=0.1) for 24 hours. **g** Luciferase activity in 293T cells transfected with IFNβ luciferase reporter, together with empty vector (EV) or different expression vectors encoding viral proteins of SARS-CoV-2 and RIG-I (2CARD). *p < 0.05, **p < 0.01. **h-i** Luciferase activity in 293T cells transfected with ISRE (**h**) or NF-κB luciferase reporter (**i**), together with empty vector (EV) or increasing amounts of M^pro^ of SARS-CoV-2. Then, the cells were transfected with cytoplasmic poly(I:C) for 12 hours. *p < 0.05, **p < 0.01. **j** Immunoassay of extracts of M^pro^-inducible A549 cells were treated with doxycycline (Dox; 200 ng·ml−1) for 24 hours, followed by SeV infection (MOI= 0.1) for indicated time points. **k** Lysates of 293T cells transfected with plasmids expressing Flag-RIG-I and HA-ubiquitin (Ub) and its indicated mutants together with empty vector or expression vectors for M^pro^, followed with SeV infection (MOI = 0.1) for 12 hours, were immunoprecipitated after SDS denaturation with anti-Flag and immunoblotted with anti-HA. **l** Immunoprecipitation and immunoassay of extracts of 293T cells transfected with Flag-TRIM25 and HA-RIG-I together with empty vector or expression vectors for M^pro^, followed with SeV infection (MOI = 0.1) for 12 hours. **p < 0.01. All the experiments are representatives of three independent biological experiments with similar results.

**Supplementary Fig. S2**

**
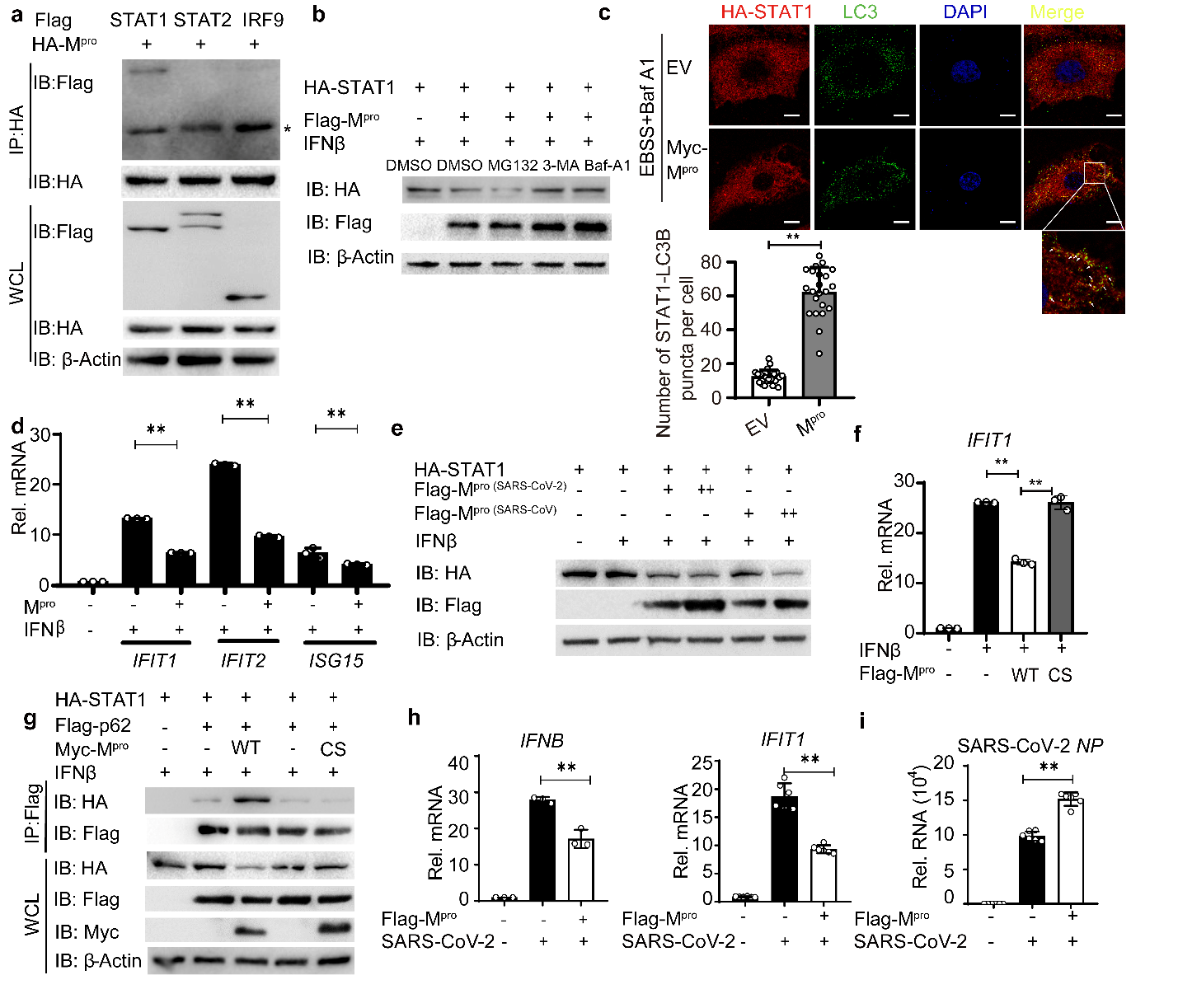
**

**Supplementary Fig. S2.** **SARS-CoV-2 M^pro^ restricts JAK-STAT signaling by targeting STAT1 for autophagic degradation.** **a** 293T cells were transfected with plasmids encoding HA-M^pro^ and Flag-tagged key proteins in STAT signaling (Flag-STAT1, Flag-STAT2, Flag-IRF9), followed by immunoprecipitating with anti-Flag beads and immunoblot analysis with anti-HA. * indicated the nonspecific bands. **b** Immunoassay of extracts of 293T cells transfected with plasmids encoding Flag-M^pro^ and HA-STAT1 followed with IFNβ treatment (1000 U·ml^−1^) for 3 hours and treated with dimethylsulfoxide (DMSO), MG132 (10 μM), 3-methyladenine (3-MA; 10mM) or Bafilomycin A1 (BafA1; 20 nM) for 6 hours before harvested. **c** Confocal microscopy analysis of HA-STAT1 and LC3B localization in Huh7 cells. (upper) Confocal microscopy analysis of Huh7 cells which were transfected with HA-STAT1 together with empty vector or Myc-M^pro^ for 24 hours, followed by BafA1 (20 nM) for 6 hours and EBSS treatment for 3 hours. Scale bars, 10 μm. (down) Quantitative analysis of the colocalization between STAT1 and LC3 (30 cells per sample). **d** Quantitative PCR with reverse transcription analysis of *IFIT1*, *IFIT2* and *ISG15* mRNA in M^pro^-inducible A549 cells. Cells were treated with doxycycline (Dox; 200 ng·ml−1) for 24 hours, followed by IFNβ treatment (1000 U·ml^−1^) for 3 hours. **p < 0.01. **e** Immunoassay of extracts of 293T cells transfected with plasmids encoding Flag-SARS-CoV-2-M^pro^ or Flag-SARS-CoV-M^pro^ and HA-STAT1 followed with IFNβ treatment (1000 U·ml^−1^) for 3 hours. **f** Quantitative PCR with reverse transcription analysis of *IFIT1* mRNA in Huh7 cells transfected with plasmids encoding wild type M^pro^ (WT) or its enzymatic inactive mutant C145S (CS) followed with IFNβ treatment (1000 U·ml^−1^) for 3 hours. **g** 293T cells were transfected with plasmids encoding wild type M^pro^ or its CS mutant as well as HA-STAT1 and Flag-p62 and treated with IFNβ (1000 U·ml^−1^) for 3 hours before harvested, followed by immunoprecipitating with anti-Flag beads and immunoblot analysis with anti-HA. **h** Quantitative PCR with reverse transcription analysis of *IFNB* and *IFIT1* mRNA in Huh7 cells transfected with empty vector or Flag-M^pro^ followed with SARS-CoV-2 infection (MOI=0.1) for 24 hours. **p < 0.01. **i** Quantitative PCR with reverse transcription analysis of SARS-CoV-2 *N protein* RNA in Huh7 cells transfected with empty vector or Flag-M^pro^ followed with SARS-CoV-2 infection (MOI=0.1) for 24 hours. **p < 0.01. All the experiments are representatives of three independent biological experiments with similar results.
